# *Spirulina platensis* Lacks Antitumor Effect against Solid Ehrlich Carcinoma in Female Mice

**DOI:** 10.1155/2015/132873

**Published:** 2015-08-20

**Authors:** Waleed Barakat, Shimaa M. Elshazly, Amr A. A. Mahmoud

**Affiliations:** ^1^Department of Pharmacology, Faculty of Pharmacy, Zagazig University, Zagazig 44519, Egypt; ^2^Department of Pharmacology, Faculty of Pharmacy, Tabuk University, Tabuk 71491, Saudi Arabia

## Abstract

Spirulina is a blue-green alga used as a dietary supplement. It has been shown to possess anti-inflammatory, antioxidant, and hepatoprotective properties. This study was designed to evaluate the antitumor effect of spirulina (200 and 800 mg/kg) against a murine model of solid Ehrlich carcinoma compared to a standard chemotherapeutic drug, 5-fluorouracil (20 mg/kg). Untreated mice developed a palpable solid tumor after 13 days. Unlike fluorouracil, spirulina at the investigated two dose levels failed to exert any protective effect. In addition, spirulina did not potentiate the antitumor effect of fluorouracil when they were administered concurrently. Interestingly, their combined administration resulted in a dose-dependent increase in mortality. The present study demonstrates that spirulina lacks antitumor effect against this model of solid Ehrlich carcinoma and increased mortality when combined with fluorouracil. However, the implicated mechanism is still elusive.

## 1. Introduction

Cancer is considered one of the leading causes of death worldwide, accounting for approximately 8.2 million deaths in 2012. Success of cancer chemotherapy is limited by drug-induced adverse effects and multidrug resistance [1, 2]. Therefore, there is a growing interest in identifying antitumor agents of natural sources, which are effective and produce fewer side effects than the conventional chemotherapeutic drugs. Actually, many of the currently used anticancer agents originate from natural sources, such as marine organisms and plants [3, 4].

Spirulina (*Arthrospira platensis*) is a blue-green alga used as a dietary supplement. It is rich in proteins, carotenoids, polyunsaturated fatty acids, vitamin B complex, vitamin E, and minerals. Additionally, it possesses other potent antioxidants such as spirulans, C-phycocyanin, and allophycocyanin [5, 6].

Spirulina phycocyanin has been shown to possess anti-inflammatory, antioxidant, and hepatoprotective properties [7–9]. In addition, different studies demonstrated the potential anticancer activity of spirulina in different experimental models [10–12].

Ehrlich ascites carcinoma (EAC) is an undifferentiated carcinoma, which is characterized by rapid proliferation, high transplantable capability, and short life span [13]. EAC bears resemblance to human tumors; therefore, the solid and the ascetic forms of this tumor are frequently utilized to evaluate the antitumor activity of different products [14, 15].

Relying on the aforementioned, the present study was conducted to evaluate the effect of spirulina against Ehrlich solid tumor induced experimentally in mice. To our knowledge, this is the first report that describes the antitumor effect of spirulina in this experimental model of EAC solid tumor. In our attempt, we compared the effects of spirulina to a reference chemotherapeutic drug, fluorouracil, and examined the effect of their combined administration as well.

## 2. Materials and Methods

### 2.1. Animals

Adult female Swiss albino mice (23 ± 2 g) were used in the current study. Mice were acclimatized for one week before starting experiments. They were housed in stainless steel cages (5 mice/cage) and kept at controlled temperature (23 ± 2°C), humidity (60 ± 10%), and light/dark (12/12 hr) cycle. Animals had free access to food and water.

#### 2.1.1. Ethical Statement

Experimental design and animal handling procedures were approved by the local authorities at the Faculty of Pharmacy, Zagazig University, Zagazig, Egypt—ECAHZU (Ethical Committee for Animal Handling at Zagazig University). Every effort was made to reduce the number of animals and their suffering.

### 2.2. Drugs

Spirulina tablets, containing 100%* Spirulina platensis* microalgae powder, were obtained from Allcura Naturheilmittel (Wertheim, Germany). 5-Fluorouracil (50 mg/mL ampoules) was obtained from Pharco Pharmaceuticals (Egypt). All other chemicals were of analytical grade. Spirulina tablets were manually crushed, ground, and then suspended in 1% gum acacia in distilled water just before administration.

### 2.3. Induction of Ehrlich Solid Tumor

On the day of induction (day 0), EAC cells were collected from the ascitic fluid of a female Swiss albino mouse bearing 8–10-day-old ascitic tumor obtained from the National Cancer Institute (Cairo, Egypt). The ascitic fluid was diluted with normal saline (1 : 10). Solid tumors were induced by intramuscular inoculation of 0.2 mL of ascitic fluid, containing approximately 2.5 × 10^6^ EAC cells, in the right thigh of the hind limb of each mouse [16].

### 2.4. Experimental Design

On the following day (day 1), EAC-bearing mice were randomly divided into six groups (*n* = 10 each) as follows.

*Group 1 (EAC).* Mice were inoculated with EAC cells and received vehicle (1% gum acacia) by oral gavage daily from day 1 to 9. 

*Group 2 (FU).* Mice were inoculated with EAC cells and received 5-fluorouracil (20 mg/kg, i.p.) daily from day 1 to 9. 

*Group 3 (SP200).* Mice were inoculated with EAC cells and received spirulina (200 mg/kg) suspended in 1% gum acacia in distilled water by oral gavage daily from day 1 to 9. 

*Group 4 (SP800).* Mice were inoculated with EAC cells and received spirulina (800 mg/kg) suspended in 1% gum acacia in distilled water by oral gavage daily from day 1 to 9. 

*Group 5 (FU/SP200).* Mice were inoculated with EAC cells and received 5-fluorouracil (20 mg/kg, i.p.) plus spirulina (200 mg/kg) suspended in 1% gum acacia in distilled water by oral gavage daily from day 1 to 9. 

*Group 6 (FU/SP800).* Mice were inoculated with EAC cells and received 5-fluorouracil (20 mg/kg, i.p.) plus spirulina (800 mg/kg) suspended in 1% gum acacia in distilled water by oral gavage daily from day 1 to 9.

Doses of spirulina and 5-fluorouracil were chosen based on previous studies [17, 18].

### 2.5. Blood Sampling, Assessment of Hematological Parameters, and Serum Preparation

On day 13, mice were anaesthetized with intraperitoneal injection of urethane (2 g/kg), and blood samples were collected from the orbital sinus using heparinized microcapillary tubes as previously described [19]. Aliquot of blood was collected from each mouse into ethylenediamine tetra-acetic acid- (EDTA-) coated tubes for the analysis of hematological parameters using an automated analyzer Swelab Alfa (Boule Medical AB, Sweden). For serum preparation, another portion of blood was collected into microcentrifuge tube and then centrifuged at 3500 rpm for 15 min. Serum was stored at −20°C and thawed just before use.

### 2.6. Determination of Tumor Weight and Volume

After blood collection, tumor-bearing thigh of each mouse was shaved; tumors were dissected, weighed, and photographed on a graph paper. Digital images were processed using ImageJ software (National Institutes of Health, USA) in order to determine the length (mm) of the major and minor axes of the tumor. Tumor volume was calculated using the following formula [16]:

(1) Tumor  volume  mm3=0.52×minor  axis×major  axis2.

### 2.7. Determination of Alanine Transaminase Activity

Serum alanine transaminase (ALT) activity was determined colorimetrically using an ALT-kit supplied by Diamond Diagnostics (Egypt), following the manufacturer's instructions. Absorbance of the final product was read using Jenway Genova spectrophotometer supplied by Bibby Scientific (Staffordshire, UK).

### 2.8. Histopathological Analysis

Specimens of tumors from different groups were excised and fixed in 10% phosphate-buffered formalin solution at room temperature. Specimens were dehydrated in graded ethanol (70–100%), cleared in xylene, and embedded in paraffin. Paraffin-embedded tissue sections (5 *μ*m thick) were prepared, mounted on slides, and kept at room temperature. Thereafter, slides were dewaxed in xylene, hydrated using graded ethanol, and stained by hematoxylin and eosin (HE) dyes. The sections were examined under light microscope and photographed with a digital camera (Canon, Japan).

### 2.9. Statistical Analysis

All data were expressed as mean ± standard error of mean (SEM). Statistical analysis was performed using GraphPad Prism software v.5 (GraphPad Software, Inc., La Jolla, CA, USA). The intergroup variation was measured by one-way analysis of variance (ANOVA) followed by Dunnett's posttest. A significant difference was assumed for values of *P* < 0.05.

## 3. Results

### 3.1. Effect on Mortality Rate

As represented in Table 1, there was no difference in mortality rate between untreated EAC tumor-bearing mice and 5-fluorouracil-treated mice. Only one mouse died from each group. On the other hand, treatment of tumor-bearing mice with spirulina (200 and 800 mg/kg) resulted in increased mortality rate reaching 50% and 30%, respectively. Similarly, combined administration of 5-fluorouracil plus spirulina (200 or 800 mg/kg) caused a noticeable dose-dependent increase in mortality rate in tumor-bearing mice reaching 50% and 90%, respectively. Therefore, further assessment of other parameters was not possible from mice that received fluorouracil plus spirulina (800 mg/kg) due to the high mortality rate (90%).

### 3.2. Effect on Tumor Weight and Volume

**Figure 1:**
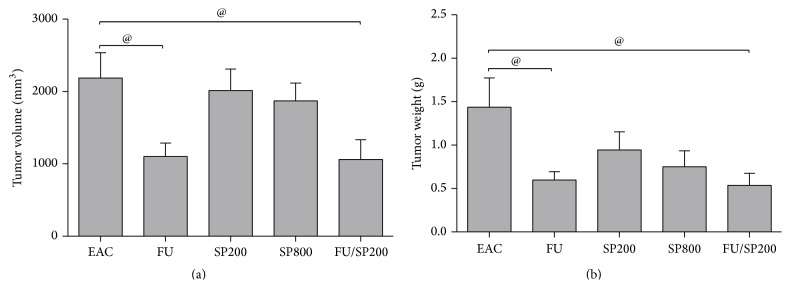
Effect of 5-fluorouracil (20 mg/kg), spirulina (200 or 800 mg/kg), and their combination on tumor volume (a) and tumor weight (b) of EAC tumor-bearing mice (*n* = 5–9). EAC: Ehrlich ascites carcinoma tumor-bearing mice; FU: Ehrlich ascites carcinoma tumor-bearing mice treated with 5-fluorouracil (20 mg/kg); SP200: Ehrlich ascites carcinoma tumor-bearing mice treated with spirulina (200 mg/kg); SP800: Ehrlich ascites carcinoma tumor-bearing mice treated with spirulina (800 mg/kg); FU/SP200: Ehrlich ascites carcinoma tumor-bearing mice treated with 5-fluorouracil (20 mg/kg) plus spirulina (200 mg/kg). Data are expressed as mean ± SEM. Statistical analysis using one-way ANOVA, followed by Dunnett's posttest. ^@^ *P* < 0.05 versus EAC group.

As depicted in Figure 1, administration of 5-fluorouracil significantly reduced tumor volume (−49.7%) and weight (−58.3%) compared to untreated EAC tumor-bearing mice (*P* < 0.05). On the other hand, treatment of tumor-bearing mice with spirulina (200 or 800 mg/kg) did not significantly reduce either the tumor volume or tumor weight. Combined administration of 5-fluorouracil plus spirulina (200 mg/kg) diminished tumor growth as indicated by the significant reduction of tumor volume (−51.5%) and tumor weight (−62.7%) compared to untreated EAC tumor-bearing mice (*P* < 0.05). There was no significant difference between mice treated with 5-fluorouracil alone or 5-fluorouracil plus spirulina (200 mg/kg) regarding tumor volume or weight.

### 3.3. Effect on Hematological Parameters

Different treatment regimens used in the present study resulted in significant alterations of some hematological parameters compared to untreated tumor-bearing mice, while other parameters were not significantly altered. Hematocrit (HCT) level was significantly reduced by the administration of 5-fluorouracil (−19.7%), spirulina 200 mg/kg (−22.7%), spirulina 800 mg/kg (−19.3%), or 5-fluorouracil plus spirulina 200 mg/kg (−27.7%) compared to untreated EAC tumor-bearing mice. No significant differences were observed between group 2, treated with 5-fluorouracil alone, and group 5, treated with 5-fluorouracil plus spirulina (200 mg/kg). Similarly, mean corpuscular volume (MCV) of red blood cells (RBCs) was significantly reduced by the administration of 5-fluorouracil (−10%), spirulina 200 mg/kg (−16.2%), spirulina 800 mg/kg (−19.7%), or 5-fluorouracil plus spirulina 200 mg/kg (−22.3%) compared to untreated EAC tumor-bearing mice. Mice received 5-fluorouracil plus spirulina (200 mg/kg) showed a further significant reduction in MCV level when compared to mice treated with 5-fluorouracil alone. From all treatment regimens, only combined administration of 5-fluorouracil plus spirulina (200 mg/kg) resulted in significant elevation of both platelet count (PT) and plateletcrit (PCT) levels when compared to either untreated EAC tumor-bearing mice or fluorouracil-treated mice (Table 2).

### 3.4. Effect on ALT

**Figure 2:**
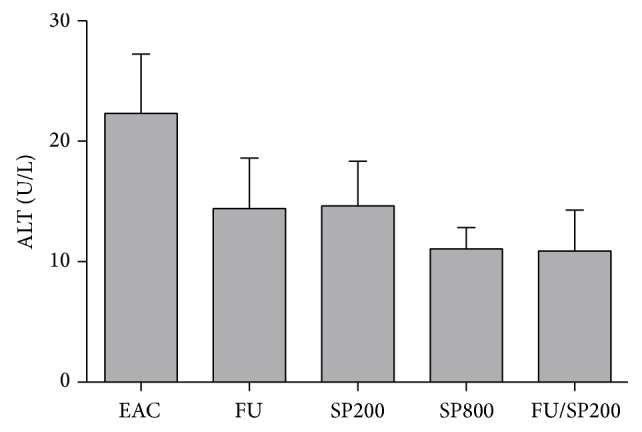
Effect of 5-fluorouracil (20 mg/kg), spirulina (200 or 800 mg/kg), and their combination on ALT level of EAC tumor-bearing mice (*n* = 4). ALT: alanine transaminase; EAC: Ehrlich ascites carcinoma tumor-bearing mice; FU: Ehrlich ascites carcinoma tumor-bearing mice treated with 5-fluorouracil (20 mg/kg); SP200: Ehrlich ascites carcinoma tumor-bearing mice treated with spirulina (200 mg/kg); SP800: Ehrlich ascites carcinoma tumor-bearing mice treated with spirulina (800 mg/kg); FU/SP200: Ehrlich ascites carcinoma tumor-bearing mice treated with 5-fluorouracil (20 mg/kg) plus spirulina (200 mg/kg). Data are expressed as mean ± SEM. Statistical analysis using one-way ANOVA, followed by Dunnett's posttest.

In our attempt to identify the cause of the increased mortality associated with the combined administration of fluorouracil and spirulina, we measured the changes in ALT levels. As shown in Figure 2, administration of different drugs resulted in reduction of ALT level when compared to untreated tumor-bearing mice. Nevertheless, this difference does not reach statistical significance. In addition, we did not notice any significant differences in ALT level between mice treated with fluorouracil alone and mice treated with spirulina alone (200 or 800 mg/kg) or fluorouracil plus spirulina (200 mg/kg).

### 3.5. Histopathological Examination

**Figure 3:**
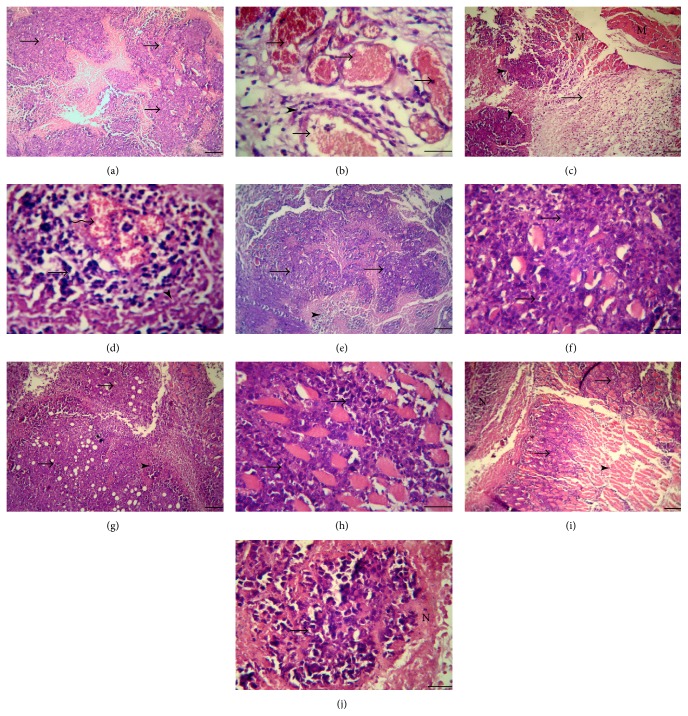
Histopathological examination of Ehrlich ascites carcinoma (EAC) solid tumor. Representative sections were obtained from (a) untreated EAC-bearing mice (arrows: infiltration of subcutaneous tissue with tumor cells), (b) untreated EAC-bearing mice (arrows: newly formed blood capillaries; arrowheads: leukocyte infiltration), (c) EAC tumor-bearing mice treated with 5-fluorouracil 20 mg/kg (arrowheads: extensive necrosis; arrow: fibrosis; M: skeletal muscles), (d) EAC tumor-bearing mice treated with 5-fluorouracil 20 mg/kg (irregular arrow: destructed blood vessels and hemorrhage), (e) EAC tumor-bearing mice treated with spirulina 200 mg/kg (arrows: extensive infiltration of the subcutaneous tissue with tumor cells), (f) EAC tumor-bearing mice treated with spirulina 200 mg/kg (arrows: tumor cells with cellular anaplasia and anisocytosis), (g) EAC tumor-bearing mice treated with spirulina 800 mg/kg (arrows: moderate infiltration of the tumor cells; arrowhead: numerous leukocyte infiltration), (h) EAC tumor-bearing mice treated with spirulina 800 mg/kg (arrows: huge numbers of tumor cells infiltrating the skeletal muscles), (i) EAC tumor-bearing mice treated with 5-fluorouracil 20 mg/kg plus spirulina 200 mg/kg (arrows: less infiltration with tumor cells; N: extensive necrosis and fibrosis), and (j) EAC tumor-bearing mice treated with 5-fluorouracil 20 mg/kg plus spirulina 200 mg/kg (arrows: islets of viable tumor cells; N: extensive necrosis). Sections were stained with HE dyes (scale bar = 50 *μ*M).

Examination of untreated tumor-bearing mice showed that EAC cells infiltrated and mostly replaced the subcutaneous tissue with necrosis of the remaining skeletal muscles. Numerous newly formed blood capillaries (neovascularization) were seen in the surrounding tissue with mild or no inflammatory response. Such tumor showed tissue architectural disarray, as well as marked degree of cellular anaplasia, pleomorphism, and anisocytosis, with nuclear vesicularity, atypicality, hyperchromasia, and mitoses. Some tumor cells were differentiated into gland-like structures surrounding a lumen containing eosinophilic material. Minimum necrotic areas with pyknosis and karyolysis, which appeared markedly in the central regions of the tumors, were noticed as well as few round-cell infiltrations and hemorrhage (Figures 3(a) and 3(b)).

On the other hand, mice treated with 5-flurourocil revealed minimal tumor cell infiltrations, extensive necrosis, and apoptosis at the margin of the tumor with destructed blood vessels and hemorrhage. Huge numbers of round cells of mostly lymphocytes and macrophages invaded the necrotic areas. Areas of the subcutaneous muscles were normal and others showed fibrous connective tissue proliferation infiltrated with leukocytes (Figures 3(c) and 3(d)).

Mice treated with spirulina alone (200 or 800 mg/kg) showed similar degree of infiltration as described with untreated tumor-bearing mice. Minimum necrosis and high vascularity of newly formed blood capillaries were also noticed. The inflammatory cell responses and necrosis in comparison with EAC were slightly increased, particularly with the higher dose of 800 mg/kg (Figures 3(e)–3(h)).

The tumor mass in mice treated with 5-flurouracil plus spirulina (200 mg/kg) was mostly necrotic (60–70%). In some parts, this was accompanied by a marked proliferation of fibrotic tissues as regeneration attempts of the subcutaneous tissue. Intense leukocyte aggregations, edema, and hemorrhage were visualized (Figures 3(i) and 3(j)). A scoring of histopathological findings is summarized in Table 3.

## 4. Discussion

Adjuvant and neoadjuvant chemotherapy represent important approaches in the management of cancer in order to reduce the recurrence following surgery or to reduce the tumor size enough to allow successful surgical removal [20, 21]. Considering various side effects caused by chemotherapeutic agents, development of new effective anticancer drugs is needed [22]. Increased attention is directed towards natural products as promising sources of anticancer therapeutic agents [23].

Spirulina is a plankton alga or cyanobacterium, which has been used as a food supplement for a long time [6, 24]. Spirulina exerts a wide array of pharmacological effects including anti-inflammatory, antioxidant, anticancer, and hepatoprotective properties [25, 26]. Therefore, in this study, we examined the effect of spirulina against Ehrlich solid tumor induced experimentally in mice.

Untreated mice inoculated with EAC cells intramuscularly in the right thigh of the hind limb developed a palpable solid tumor in 13 days following inoculation. This is consistent with other previous studies that used the same model [18, 27]. Administration of 5-fluorouracil (20 mg/kg) for 9 consecutive days starting from day 1 following EAC cells inoculation significantly reduced both tumor volume and tumor weight compared to untreated EAC tumor-bearing mice. On the other hand, administration of spirulina alone (200 or 800 mg/kg) did not significantly alter the tumor size compared to untreated EAC tumor-bearing mice. Although spirulina showed a dose-dependent tendency towards reducing the tumor volume, however, such change was not significant. These results reveal that spirulina alone lacks antitumor effect in this experimental model. We assume that this lack of activity is not attributed to the use of small doses of spirulina because even at a dose of 800 mg/kg no effect was observed. In addition, we rule out poor oral absorption of spirulina as a possible cause of the lack of the antitumor effect based on previous reports showing good bioavailability of different constituents of spirulina including carotenoids [28–30], iron [31], and proteins [32]. This lack of effect can be viewed also at the histopathological level. Tumor specimen from mice treated with spirulina alone (200 or 800 mg/kg) showed EAC cells that infiltrated and mostly replaced the subcutaneous tissue. The observed degree of infiltration was similar to that found in specimens from untreated tumor-bearing mice. In addition, minimum necrosis was noticed in EAC tumor-bearing mice treated with spirulina alone at both dose levels (10–15%), which is very close to untreated mice (8–12%).

The lack of the antitumor effect of spirulina in our model does not conform to some other reports that described a potential anticancer activity of spirulina in other experimental models. Yogianti et al. [33] showed that spirulina exerts antitumor effects against UVB-induced skin tumor development in mice. In addition, it has been reported that spirulina exerts a chemopreventive effect against 7,12-dimethylbenz[a]anthracene-induced breast carcinogenesis [34] and against dibutyl nitrosamine-induced liver cytotoxicity and carcinogenesis [12] in rat. The discrepancy between these data and our results might be attributed to differences in the experimental conditions, the model of the solid tumor used, or the limited ability of spirulina to penetrate through Ehrlich solid tumor tissue and to reach all of the tumor cells in a potentially lethal concentration. The latter has been recognized as an important cause of anticancer drug resistance [35].

On the other hand, mice treated with 5-flurourocil alone showed minimal tumor cell infiltrations and extensive necrosis (50–60%). To test whether spirulina can at least potentiate the antitumor effect of fluorouracil, we examined the effect of their combined administration. Fluorouracil plus spirulina (200 mg/kg) significantly reduced both tumor volume and weight compared to untreated tumor-bearing mice; however, there were no significant differences in tumor volume or weight compared to mice treated with fluorouracil alone. In addition, histopathological examination revealed that the tumor mass in mice treated with both 5-flurouracil and spirulina (200 mg/kg) was mostly necrotic (60–70%) comparable to fluorouracil alone.

Interestingly, we observed a substantial, dose-dependent increase in mortality rate in mice treated with fluorouracil plus spirulina at a dose of 200 mg/kg (5 out of 10) and fluorouracil plus spirulina at a dose of 800 mg/kg (9 out of 10). We assume that this toxic effect is not attributed to EAC-tumor development* per se* or fluorouracil administration* per se* because only one animal died from group 1 (EAC-bearing mice) and group 2 (fluorouracil-treated mice). Therefore, it seems that the combined administration of fluorouracil and spirulina concurrently is responsible for such toxic effect.

We tried further to explain the mechanism implicated in the increased mortality by examining the effect on some hematological parameters and ALT level to see if this combination can cause acute hemotoxicity or fulminant hepatic damage. No significant alterations in complete blood count (CBC) were observed except for reduction in mean corpuscular volume (MCV) and increase in platelet count (PT) and plateletcrit (PCT) in mice treated with fluorouracil plus spirulina (200 mg/kg) when compared to mice treated with fluorouracil alone. In addition, there was no significant difference in ALT level between both groups. These results rule out hemotoxicity or hepatotoxicity as possible causes of increased mortality.

Depending on the present available results, the underlying mechanism of increased mortality is yet elusive; however, some postulations are worth mentioning. Different reports described a hypotensive effect associated with the administration of either 5-fluorouracil [36–38] or spirulina [39]. It is likely that combined administration of fluorouracil and spirulina, particularly at high dose level of 800 mg/kg, could enhance the reduction of blood pressure and might result in cardiovascular collapse. On the other hand, spirulina may have increased fluorouracil toxicity because of possible inhibition of the activity of dihydropyrimidine dehydrogenase, the enzyme that catalyzes the first rate-limiting step of fluorouracil degradation [40]. This assumption is based on a previous study reporting that spirulina resulted in inhibition of activities of some hepatic cytochrome P450 enzymes [41]. Although we could not find any report in the literature describing a direct relation between spirulina and dihydropyrimidine dehydrogenase, a possible effect could still be expected. Certainly, these assumptions need to be thoroughly investigated.

In conclusion, the present study describes the lack of antitumor activity of spirulina in EAC tumor-bearing mice model. In addition, spirulina administered simultaneously with fluorouracil did not enhance the antitumor activity of the later but rather resulted in increased dose-dependent mortality. In light of the present results, the mechanism of spirulina-induced mortality is not well understood. Although spirulina has been shown to possess anticancer effects in other models, the present study shows that it might not be a suitable therapeutic alternative for conventional chemotherapeutic agents in all settings such as EAC tumor-bearing mice model. In addition, spirulina might be not very safe, particularly when administered with other drugs such as fluorouracil. Therefore, we recommend that spirulina, or even other natural products, should be used cautiously and that possible interactions with other coadministered drugs should be monitored carefully.

## Figures and Tables

**Table 1:** Effects of 5-fluorouracil (20 mg/kg), spirulina (200 or 800 mg/kg), and their combination on the survival of EAC tumor-bearing mice.

Group	Number tested	Survivors/total mice	% Mortality
EAC^a^	10	9/10	10%
FU^b^	10	9/10	10%
SP200^c^	10	5/10	50%
SP800^d^	10	7/10	30%
FU/SP200^e^	10	5/10	50%
FU/SP800^f^	10	1/10	90%

^a^EAC: Ehrlich ascites carcinoma tumor-bearing mice; ^b^FU: Ehrlich ascites carcinoma tumor-bearing mice treated with 5-fluorouracil (20 mg/kg); ^c^SP200: Ehrlich ascites carcinoma tumor-bearing mice treated with spirulina (200 mg/kg); ^d^SP800: Ehrlich ascites carcinoma tumor-bearing mice treated with spirulina (800 mg/kg); ^e^FU/SP200: Ehrlich ascites carcinoma tumor-bearing mice treated with 5-fluorouracil (20 mg/kg) plus spirulina (200 mg/kg); ^f^FU/SP800: Ehrlich ascites carcinoma tumor-bearing mice treated with 5-fluorouracil (20 mg/kg) plus spirulina (800 mg/kg).

**Table 2:** Effects of 5-fluorouracil (20 mg/kg), spirulina (200 or 800 mg/kg), and their combination on hematological parameters of EAC tumor-bearing mice.

	EAC	FU	SP200	SP800	FU/SP200
RBC (10^12^/L)	7.6 ± 0.27	6.8 ± 0.27	6.9 ± 0.77	7.7 ± 0.48	7.1 ± 0.36
HGB (g/L)	11.4 ± 0.37	9.8 ± 0.41	10.1 ± 1.1	10.8 ± 0.65	10.3 ± 0.45
HCT (%)	46.8 ± 1.3	37.6 ± 1.5^a^	36.1 ± 4.1^a^	37.8 ± 2.3^a^	33.8 ± 1.5^a^
MCV (fL)	61.5 ± 0.9	55.3 ± 2.3^a^	51.6 ± 0.6^a^	49.4 ± 1.5^a^	47.8 ± 1.5^ab^
PT (10^9^/L)	737 ± 64	959 ± 102	947 ± 39	806 ± 80	1403 ± 160^ab^
PCT (%)	0.47 ± 0.05	0.69 ± 0.08	0.59 ± 0.04	0.54 ± 0.07	1.0 ± 0.12^ab^
WBC (10^9^/L)	8.1 ± 0.81	6.2 ± 1.4	10.2 ± 2.1	10.7 ± 2.0	8.9 ± 3.3
LYM (10^9^/L)	7.3 ± 0.75	4.9 ± 0.97	7.8 ± 1.6	7.3 ± 1.3	6.9 ± 2.3
GRA (10^9^/L)	0.28 ± 0.08	0.41 ± 0.17	0.48 ± 0.19	0.55 ± 0.19	0.53 ± 0.27

Values are expressed as mean ± SEM (*n* = 5–9). EAC: Ehrlich ascites carcinoma tumor-bearing mice; FU: Ehrlich ascites carcinoma tumor-bearing mice treated with 5-fluorouracil (20 mg/kg); SP200: Ehrlich ascites carcinoma tumor-bearing mice treated with spirulina (200 mg/kg); SP800: Ehrlich ascites carcinoma tumor-bearing mice treated with spirulina (800 mg/kg); FU/SP200: Ehrlich ascites carcinoma tumor-bearing mice treated with 5-fluorouracil (20 mg/kg) plus spirulina (200 mg/kg). RBC: red blood cell count; HGB: hemoglobin; HCT: hematocrit; MCV: mean corpuscular volume; PT: platelet count; PCT: plateletcrit; WBC: white blood cell count; LYM: lymphocyte count; GRA: granulocyte count. Statistical analysis using one-way ANOVA, followed by Dunnett's posttest. ^a^ *P* < 0.05 versus EAC and ^b^ *P* < 0.05 versus FU.

**Table 3:** Histopathology scoring of tumor-bearing mice treated with 5-fluorouracil (20 mg/kg), spirulina (200 or 800 mg/kg), and their combination.

	Necrosis	Neovascularization	Leukocytes	Fibrosis
EAC^a^	8–12%	+++	+	−
FU^b^	50–60%	+	+++	++
SP200^c^	10–15%	+++	++	−
SP800^d^	10–15%	+++	++	−
FU/SP200^e^	60–70%	+	+++	+++

^a^EAC: Ehrlich ascites carcinoma tumor-bearing mice; ^b^FU: Ehrlich ascites carcinoma tumor-bearing mice treated with 5-fluorouracil (20 mg/kg); ^c^SP200: Ehrlich ascites carcinoma tumor-bearing mice treated with spirulina (200 mg/kg); ^d^SP800: Ehrlich ascites carcinoma tumor-bearing mice treated with spirulina (800 mg/kg); ^e^FU/SP200: Ehrlich ascites carcinoma tumor-bearing mice treated with 5-fluorouracil (20 mg/kg) plus spirulina (200 mg/kg). (−) none; (+) mild; (++) moderate; (+++) severe; (++++) more severe.
